# Comorbidities against Quality Control of VKA Therapy in Non-Valvular Atrial Fibrillation: A French National Cross-Sectional Study

**DOI:** 10.1371/journal.pone.0119043

**Published:** 2015-03-19

**Authors:** Agnes Rouaud, Olivier Hanon, Anne-Sophie Boureau, Guillaume Gilles Chapelet, Laure de Decker

**Affiliations:** 1 Department of Geriatrics, EA 1156–12, Nantes University Hospital, Nantes, France; 2 Department of Geriatrics, Broca Hospital, Public Hospital of Paris, Paris, France; University of Bologna, ITALY

## Abstract

**Background:**

Given the prevalence of non-valvular atrial fibrillation in the geriatric population, thromboembolic prevention by means of vitamin K antagonists (VKA) is one of the most frequent daily concerns of practitioners. The effectiveness and safety of treatment with VKA correlates directly with maximizing the time in therapeutic range, with an International Normalized Ratio (INR) of 2.0-3.0. The older population concentrates many of factors known to influence INR rate, particularly concomitant medications and concurrent medical conditions, also defined as comorbidities.

**Objective:**

Determine whether a high burden on comorbidities, defined by a Charlson Comorbidity Index (CCI) of 3 or greater, is associated a lower quality of INR control.

**Study-Design:**

Cross-sectional study.

**Settings:**

French geriatric care units nationwide.

**Participants:**

2164 patients aged 80 and over and treated with vitamin K antagonists.

**Measurements:**

Comorbidities were assessed using the Charlson Comorbidity Index (CCI). The recorded data included age, sex, falls, kidney failure, hemorrhagic event, VKA treatment duration, and the number and type of concomitant medications. Quality of INR control, defined as time in therapeutic range (TTR), was assessed using the Rosendaal method.

**Results:**

487 patients were identified the low-quality control of INR group. On multivariate logistic regression analysis, low-quality control of INR was independently associated with a CCI ≥3 (OR = 1.487; 95% CI [1.15; 1.91]). The other variables associated with low-quality control of INR were: hemorrhagic event (OR = 3.151; 95% CI [1.64; 6.07]), hospitalization (OR = 1.614, 95% CI [1.21; 2.14]).

**Conclusion:**

An elevated CCI score (≥3) was associated with low-quality control of INR in elderly patients treated with VKA. Further research is needed to corroborate this finding.

## Introduction

Non-valvular atrial fibrillation (NVAF) grows more prevalent with age, particularly after 60 [1]. The incidence of non-valvular atrial fibrillation affects 8 percent of patients 80 years of age or older, and 20 percent of patients over 90 [2]. Thromboembolic disorders such as stroke rank among the most frequent complications in NVAF. Aging is one of the leading independent risk factors demonstrated to increase thromboembolic disorders in NVAF, particularly after the age of 75 [3]. These elements make older patients a special target group for preventive thromboembolic treatments. Traditional oral anticoagulation therapy by vitamin K antagonist (VKA) is widely used and has demonstrated efficacy in preventing such outcomes [4]. The rate of anticoagulation obtained through VKA is evaluated by International Normalized Ratio (INR). The effectiveness and safety of VKA are highly correlated to maintaining INR in a narrow therapeutic window [5,6]. Indeed, oral anticoagulation can lead to adverse outcomes (bleeding or thromboembolic events) directly related to INR outside the therapeutic window [5–7]

The most widely recommended approach for evaluating the quality and safety of anticoagulation is to estimate the percentage of time in therapeutic range (TTR), that is to say the time spent within the therapeutic international normalized ratio limits [8,9]. Despite close supervision and daily adaptation of drug dosages, in observational studies only 50% of the patients remain within the therapeutic window [10,11]. Most studies have evaluated which factors are associated with high-quality control of INR [12–20]. But in order to prevent adverse effects while maintaining the effectiveness of a treatment in daily clinical practice, it would appear to be more important to identify which factors can be associated with low-quality control of INR.

It is well established that the dose response for VKA is affected by significant inter- and intra-individual factors such as age, concomitant use of others drugs [21], genetic polymorphisms [22,23], nutritional status and vitamin K intake [21] and some acute or chronic diseases [24]. Older patients have several prescribing challenges with additional barriers to anticoagulation control. Indeed, they combine concomitant medications and concurrent medical conditions, also defined as comorbidities, known to disrupt the stability of anticoagulation by VKA (congestive heart failure [25], hyperthyroidism illness [26], malnutrition [27], fever [24], etc.). For each of these medical conditions, most of the studies have individually shown an association with an INR beyond the therapeutic range. The hypothetical interaction between multiple concurrent medical conditions, or comorbidities, and INR has not been the subject of many analyses. Actually, no study has evaluated the possible interaction between the burden of comorbidities, estimated by CCI, and quality of INR control estimated by TTR.

Our hypothesis is that a high burden of comorbidities, calculated by CCI, is associated with a lower quality of INR control. The aim of this study was to identify whether the CCI is associated with low-quality INR control.

## Methods

### Standard Protocol Approvals, Registrations and Patient Consents

The study was conducted in accordance with the ethical standards set forth in the Helsinki Declaration (1983). The entire study protocol was approved by the local Ethical Committee of Nantes (Groupe Nantais d’Ethique dans le Domaine de la Santé—GNEDS, France), and the study is in compliance with the Strengthening the Reporting of Observational Studies in Epidemiology statement guidelines. The institutional review board waived the need for written informed consent from the participants. Waiving of consent was authorized for this study according to French law. All recording patient’s data were anonymized prior to analysis.

### Participants

A cross-sectional survey conducted by the French Society of Geriatrics and Gerontology (SFGG) included inpatients on June 21, 2011, who were treated with VKA in atrial fibrillation [28]. Eligible patients were 80 years of age or older and present in a French geriatric care unit (geriatric acute-care service, post-acute, acute care and rehabilitation, nursing home). Patients were excluded if they took VKA to treat venous thrombosis or pulmonary embolism, or to prevent thromboembolic events in cardiac valvular pathologies or other pathologies (e.g. antiphospholipid antibody syndrome).

### Clinical assessment

A standardized questionnaire was sent by email to 1500 practitioners. Physician-reported responses were received from 482 practitioners working a geriatric care unit on June 21, 2011. Data collection included factors thought to be associated with quality of INR control as suggested by previous studies [13–19]. For each patient, the following information was collected: sex, age, weight, plasma creatinine, time since instauration of anticoagulation by VKA (more than one year, between three months and a year, and less than three months), history of hemorrhagic event, number and types of current co-medications, type of care structure unit. The last two consecutive bioassays of INR were collected to calculate time in therapeutic range. Falls were considered as significant if the patient fell twice or more during the year preceding data collection [29]. A kidney injury was defined based on the glomerular filtration rate computed using the Cockcroft-Gault formula. Patients were also separated into three groups: severe kidney failure (<30 ml/min), moderate kidney failure (>30 ml/min and <60 ml/min), and no kidney failure (up to 60 ml/min). The type of care structure unit was separated into two groups: hospitalization (geriatric acute-care service or post-acute and rehabilitation) or institutionalization in a nursing home. Current concomitant medications known to interact with VKA by an INR modification were: antifungals, antibiotics, acetaminophen, proton pump inhibitors (PPI), serotonin reuptake inhibitors (SRI) and statin [21].

The burden of comorbidities was evaluated using the Charlson Comorbidity Index (CCI) [30]. Comorbidities were defined as acute or chronic co-existing diseases referring to an index pathology at the time of the study [31]. The CCI is widely used in studies of older patients and demonstrates strong interjudge reliability and good reproducibility [32–34]. The CCI score is the sum of the weightings for all the patient’s conditions. We obtained the total score by adding the points for all 19 relevant pathological statuses. The relative-risk of death increased by 2.3 for each increment of the Charlson Comorbidity Index. The total score is a continuous variable ranging from 0 to 30. A CCI score ≥3 has been associated with severe comorbid conditions, a high rate of mortality [30]. A CCI score ≥3 is also the usual distribution of the CCI score in the older population, particularly after 80 years old [35,36]. This value was set as the threshold to separate patients into two groups for purposes of this analysis. Patients were thus deemed to suffer from no severe burden of comorbidity if CCI <3 and from a severe burden of comorbidity if CCI ≥3.

Quality of INR control can be evaluated based on time in the therapeutic range. The time in therapeutic range (TTR) can be assessed in three different ways. First, there is the traditional method that estimates the number of INR strictly in the therapeutic range according to the total number of INR controls [37]. Second is the cross-sectional method: the appraiser chooses a date index and identifies all the INR at this time that are in the therapeutic range based on the entire INR control for this day [37]. Finally, there is the Rosendaal method [38]: a percentage of time in therapeutic range, with the INR-specific person-time calculated by incorporating the frequency of INR measurements and their actual values while assuming changes between consecutive INR measurements are linear over time. In our study, we chose to evaluate INR stability using the Rosendaal method. This choice was guided by two constraints: first, only two measurements of INR were available; and secondly, the Rosendaal method is among those used most frequently today [8,9,39]. We then chose to treat the TTR in a binary variable according to the distribution of the TTR in our population determined by the Rosendaal method. In addition, the first group, “high-quality control of INR,” consisted of patients with a TTR of between 25% and 100%. The second group, “low-quality control of INR,” was comprised of patients with a TTR of between 0% and 24%.

### Statistics

Patients’ baseline characteristics were summarized using means and standard deviations, or frequencies and percentages, as appropriate. Normality of the data distribution was evaluated using the Skewness-Kurtosis test. As the number of observations was >40 for each group, no transformations were applied to the variables of interest. Patients were separated into two groups based on the stability or not of INR. Between-group comparisons were performed using an independent sample *t*-test or chi-square test, as appropriate. Univariate and multiple linear logistic analyses were performed to identify the association between INR stability (dependent variable) and a CCI score ≥3 (independent variable) and adjusted for the patients’ baseline characteristics. P-values ≤0.05 were taken to be indicative of statistical significance. All statistics were computed using SAS (release 9.1.3.; SAS Institute, Cary, NC).

## Results

We included 2164 patients in this survey. The low-quality control of INR group consisted of 487 patients (22.5%), while the high-quality control of INR group numbered 1677 patients (77.5%). The low-quality control of INR group consisted mainly of women (n = 331; 68%), mean age 87.1 ± 5.1 years old. They took a mean of 8.9 concomitant medications. The delay of prescription of VKA was up to 12 months (n = 410, 81.7%). Most of them were suffering from moderate kidney failure (50.7%), and more than a quarter of them had severe kidney failure (27.5%). Comparisons of the characteristics of the two groups can be seen in Table 1. Patients with low-quality control of INR experienced more hemorrhagic events (p = 0.018), were more frequently hospitalized than in nursing home (p<0.001), frequently took serotonin reuptake inhibitors (p = 0.002), proton pump inhibitors (p = 0.040) and antifungals (p = 0.035), suffered from severe kidney failure (p = 0.040), had VKA treatment longer than one year (p = 0.028) or less than three months (p = 0.010) and had fallen twice or more during the previous year (p = 0.031) than patients with high-quality control of INR. There were no other significant differences observed between the two groups with respect to their clinical characteristics.

**Table 1:** Characteristics of patients according to low-quality control of INR (n = 2164).

Characteristics	Low-quality control of INR		p-value*
	yes	no	
	(n = 487)	(n = 1677)	
Age (years), mean ±SD	87.1 ± 5.1	86.9 ± 4.6	p = 0.370
Female sex, n (%)	331 (68)	1158 (69.4)	p = 0.655
Hemorrhagic event, n (%)	19 (4)	33 (2)	**p = 0.018**
Hospitalization, n (%)	215 (44.5)	655 (30.9)	**p < 0.001**
Number of concomitant medications, mean ±SD	8.9 ± 3.0	8.8 ± 3.0	p = 0.699
Type of concomitant medications			
Antifungals, n (%)	25 (5.2)	51 (3)	**p = 0.035**
Antibiotics, n (%)	51 (10.5)	131 (7.8)	p = 0.064
Acetaminophen, n (%)	283 (58.4)	984 (58.8)	p = 0.875
PPI, n (%)	215 (44.6)	655 (39.3)	**p = 0.040**
SRI, n (%)	121 (25.1)	544 (32.5)	**p = 0.002**
Statin, n (%)	97 (20.1)	364 (21.8)	p = 0.450
Associated comorbidities			
CCI ≥ 3, n (%)	279 (57.3)	882 (52.6)	p = 0.071
Falls twice or more in a year, n (%)	138 (28.8)	398 (23.9)	**p = 0.031**
Kidney failure			
CrCl < 30 ml/min, n (%)	134 (27.5)	384 (22.9)	**p = 0.040**
30 < CrCl < 60 ml/min, n (%)	247 (50.7)	917 (54.7)	p = 0.134
CrCl > 60 ml/min, n (%)	106 (21.8)	376 (22.4)	p = 0.805
VKA treatment duration			
≤ 3 month, n (%)	37 (9)	80 (5.4)	**p = 0.010**
Between 3 and 12 months, n (%)	38 (7.8)	124 (8.4)	p = 0.619
≥ 12 months, n (%)	410 (81.7)	1277 (86.2)	**p = 0.028**

* based on independent samples chi-square or *t-*test as appropriate with *P* significant ≤ 0.05; significant *P*-value (i.e. ≤0.05) indicated in bold

INR: international normalized ratio; PPI: proton pump inhibitors; SRI: Serotonin Reuptake Inhibitors; CrCl: creatinine clearance according to the Cockcroft-Gault formula; VKA: vitamin K antagonist.

Under the univariate model presented in Table 2, low-quality control of INR was associated with hemorrhagic events (p = 0.016), been hospitalized (p < 0.001), administration of proton pump inhibitors (p = 0.037), administration of serotonin reuptake inhibitors (p = 0.002), experienced falls twice or more in a year (p = 0.029); having started VKA treatment less than a month prior (p = 0.008), having had VKA treatment for longer than one year (p = 0.025) and suffering from severe kidney failure (p = 0.036). No statistical differences were found between the two groups in terms of their age, sex, and number of co-medications, or CCI ≥3. There were also no differences between groups regarding the administration of antifungal, antibiotics, acetaminophen or statin.

**Table 2:** Uni- and multivariate logistic regression models showing the association between low-quality control of INR and Charlson Comorbidity Index ≥ 3 adjusted for clinical characteristics (n = 2164).

Characteristics	Unadjusted model			Fully adjusted model*		
	OR	CI 95%	p-value	OR	CI 95%	p-value
Age	1.010	[0.99; 1.03]	p = 0.370	1.014	[0.99; 1.04]	p = 0.305
Female sex	0.948	[0.76; 1.18]	p = 0.634	0.984	[0.75; 1.28]	p = 0.907
Hemorrhagic event	**2.023**	[1.14; 3.60]	**p = 0.016**	**3.151**	[1.64; 6.07]	**p = 0.001**
Number of concomitant medications	1.007	[0.97; 1.04]	p = 0.699	0.988	[0.95; 1.03]	p = 0.576
Hospitalization	**1.795**	[1.46; 2.21]	**p < 0.001**	**1.614**	[1.21; 2.14]	**p = 0.001**
Associated treatments						
Antifungals	**1.731**	[1.06; 2.82]	**p = 0.028**	1.605	[0.86; 3.01]	p = 0.140
Antibiotics	1.385	[0.98; 1.95]	p = 0.061	1.399	[0.91; 2.14]	p = 0.122
Acetaminophen	0.981	[0.80; 1.20]	p = 0.854	0.930	[0.72; 1.20]	p = 0.577
PPI	**1.244**	[1.01; 1.53]	**p = 0.037**	1.201	[0.93; 1.55]	p = 0.159
SRI	**0.696**	[0.55; 0.87]	**p = 0.002**	0.780	[0.59; 1.02]	p = 0.074
Statin	0.905	[0.70; 1.16]	p = 0.438	0.802	[0.59; 1.10]	p = 0.168
Associated comorbidities						
CCI ≥ 3	1.209	[0.99; 1.48]	p = 0.068	**1.487**	[1.15; 1.91]	**p = 0.002**
Falls twice or more in a year	**1.288**	[1.03; 1.62]	**p = 0.029**	1.263	[0.957–1.67]	p = 0.099
Kidney failure						
CrCl <30 ml/min	**1.278**	[1.02; 1.61]	**p = 0.036**	Ref^†^	-	-
30 < CrCl < 60 ml/min	0.853	[0.70; 1.04]	p = 0.123	0.759	[0.56; 1.02]	p = 0.068
CrCl >60 ml/min	0.953	[0.75; 1.23]	p = 0.760	0.937	[0.65; 1.35]	p = 0.730
VKA treatment duration						
≤ 3 months	**1.738**	[1.16; 2.61]	**p = 0.008**	Ref^†^	-	-
Between 3 and 12 months	1.109	[0.75; 1.62]	p = 0.595	0.673	[0.37; 1.21]	p = 0.188
≥ 12 months	**0.717**	[1.54; 0.96]	**p = 0.025**	0.634	[0.39; 1.02]	p = 0.059

*: based on logistic regression model with *P* significant ≤0.05; Significant *P*-value (i.e. ≤0.05) or OR indicated in bold

^†^: Score = 1 used as reference level

OR: Odds ratio; CI: Confidence Interval; INR: international normalized ratio; PPI: proton pump inhibitors; SRI: Serotonin Reuptake Inhibitors; CCI: Charlson Comorbidity Index; CrCl: creatinine clearance according to the Cockcroft-Gault formula; VKA: vitamin K antagonist

On multivariate logistic regression analysis, low-quality control of INR was independently associated with a CCI ≥3 (OR = 1.487; 95% CI [1.15; 1.91]). The other variables associated with low-quality control of INR were: hemorrhagic event (OR = 3.151; 95% CI [1.64; 6.07]), hospitalization (OR = 1.614, 95% CI [1.21; 2.14]).

## Discussion

Our finding shows that three variables appear to be associated with low-quality control of INR. First, a high burden of comorbidities (CCI ≥3; (OR = 1.487; 95% CI [1.15; 1.91]); second, hemorrhagic event (OR = 3.151; 95% CI [1.64; 6.07]); and third, hospitalization (OR = 1.614, 95% CI [1.21; 2.14]).

One of the most interesting results is that a high burden of comorbidities, defined as a CCI ≥3, is associated with a low-quality control of INR. Our study, using a validated and frequent used comorbidity score, is consistent with a study by Witt et al. showing that every 1-point increase in Chronic Disease Score (CDS) is associated with an 8% reduction in the likelihood of having a good-quality control of INR [15]. Moreover, the recent study investigated by Menzin et al. suggests this higher variability of INR in patients with a high burden of comorbidity. Indeed, they found that INR among patients who were older and with a Charlson Comorbidity Index ≥4 was more likely to remain uncorrected within one day following fresh frozen plasma administration [40].

As we could expect, hemorrhagic event is associated with lower quality control of INR. The association between bleeding risk and INR level is well known and clearly linked to an INR out of therapeutic range [5]. Beyond obvious evidence, this finding may encourage practitioners to be very vigilant about every hemorrhagic event that may raise questions about the safety and efficacy of VKA treatment.

Another interesting point of this study is that hospitalization is a critical period for good quality control of INR in patients receiving VKA. Our result is consistent with current data of the international literature that had however suggest a lower quality-control of INR in hospitalized patients [13,16]. In Van Walraven *and al* study, the proportion of day in therapeutic range of patients who were hospitalized decreased by 15% compared to patients who were not hospitalized (adjusted RR = 0.85, 95% CI [0.83; 0.87] [13]. In Rose *and al* study, they found a decrease TTR with increasing numbers of hospitalization [16]. Several factors may explain this association. First, a hospitalization event entails numerous changes in diet, lifestyle, health status itself, and concomitant drugs especially introduce acute illnesses that can disturb the management of VKA treatment, as has been noted in earlier studies [24]. Second, an inadequate INR could require hospitalization due to bleeding or a thromboembolic event directly linked to an INR out of therapeutic range [5,6].

Contrary to actual data, we found no association between low-quality control of INR and VKA duration treatment. Indeed, the first three months of VKA treatment constitute a critical period and are already related to INR out of therapeutic range [41]. Costa *and al* suggested that warfarin initiation lasting longer than two months is a predictor of good anticoagulation quality [18]. This result may be due to the very low proportion of our sample of population recently having VKA.

With regard to concomitant medications known to interact with INR [21], no association with low-quality control of INR was noticed in the multivariate analysis with reference to statin, proton pump inhibitors (PPI), serotonin reuptake inhibitors (SRI), acetaminophen, antibiotics and antifungals. Several factors may lead to this result. First, it is difficult to explain potential drug interactions with VKA, because many of them are only case reports, small cohorts or poor-quality studies with divergent outcomes [21]. Second, interactions with VKA and each molecule in different drug families produce very different expressions of INR level (e.g. omeprazole potentiates INR level, while pantoprazole has no effect [21]). Pooling every molecule in a single drug family may offset individual effects on INR level.

Contrary to the findings of Rose *et al*., we found no association between the number of concomitant medications and low-quality control of INR [16]. This result may be explained by a methodological approach to examine the association of comorbidities with low-quality control of INR. First, we choose to lead the number of concomitant medications as a continuous variable while Rose *et al*. use stratification. Second, in a previous study in which low-quality of INR control was linked to a high number of concomitant medications [16], comorbidities were not including in logistic regression models. But the burden of comorbidities has been associated with the number of concomitant medications [42]. The fact that we found that the comorbidity burden was associated with low-quality control of INR, regardless of the number of concomitant medications, underlines a specific effect.

Contrary to the current findings in the international literature, we found no association between age, gender, falls, chronic kidney failure and low-quality control of INR. Where age is concerned, growing older seems to yield greater quality control of INR [15,16]. In terms of gender, the data in the current literature gave men an advantage, with greater quality control of anticoagulation [13,15,16,20]. The potential association between falls and having a low-quality control of INR has not been study yet, even if sarcopenia and frailty already known to affect INR values [27]. And for patients suffering from chronic kidney failure, current data showed that patients with kidney failure spent significantly less time in therapeutic range [13,16,17]. Two elements can explain our contradictive results. First, all the precedent studies relied on a very different methodology, particularly where the definition of INR stability is concerned. Second, the population included in all the precedent studies was younger than ours, and this means fewer confounding factors—notably, comorbidities. Further prospective studies must be conducted to clarify the relationships among age, gender, chronic kidney failure and quality of INR control.

The present study had some limitations. A reporting bias may be included in the dataset. Indeed, the accuracy and completeness of our data were entirely reliant upon physicians’ declarations. But the questionnaire was designed to limit variability in readers’ interpretations by asking only factual data.

The definition of quality of INR control using the Rosendaal method could influence our results. Indeed, TTR calculated by linear extrapolation associated with an increased proportion of stable INR, compared to other methods for estimating TTR [11]. Moreover, this method is a mathematical extrapolation that only predicts TTR and reports real INR values only in part. Only two bioassays were available to determine TTR with no exclusion of very low or very high INR. Rosendaal *and al*. confirmed in their validation study, that extreme values of INR lead to an overestimation of the TTR [38]. They also prevent about great variability of the linear extrapolation between subsequent measurements, particularly after dose adjustment has been made. Variations of INR values are frequent with recent hospitalization, VKA’s dose adjustment at initiation of the treatment or medication change. We should have excluded patients with extremes values of the INR and a greater number of INR would have been beneficial to limit this potential bias. All these elements can explain why a large share of our patients sorted into the high-quality control of INR group. In our sample, the TTR distribution appears to be similar to actual data of the international literature, with 22.5% of our population having TTR between 0% and 25%. Morgan and al. noticed in their study that 20.5% of their 2 235 patients treated by warfarin had a TTR between 0% and 30% [7].

The threshold used in our study to define low-quality control of INR was determined by the statistical distribution of our population. The high-quality control of INR group was strictly defined as being in therapeutic range more than 25% of the time, while the low-quality control of INR group had a TTR of less than 25%. This is open to discussion: is it clinically pertinent to attribute a high-quality control of INR if a patient has a TTR of just above 25%? This threshold largely led to underestimation of the low-quality control-of-INR group, contrary to current French and international data [10,11]. Still, the Rosendaal method was the only available methodology adapted to our design study. Moreover, each study evaluating VKA dose response chose a different method to assess the quality of INR control and different thresholds [13–20].

Completeness is impossible when it comes to factors to explain the individual dose response of VKA, but the best accuracy in clinical studies is needed. Despite our efforts, a number of confounding factors that can influence response to VKA have not been filled, such as malnutrition, genetic factors, dietary vitamin K intake, etc. This may alter statistical results as well, but our large sample of elderly and the number of variables included prevent this bias.

## Conclusion

This study highlights the fact that an elevated CCI score (≥3) was associated with low-quality control of INR in older patients treated by VKA. Older patients with numerous comorbid conditions may require greater attention by practitioners or may receive innovative management strategies to achieve acceptable levels of anticoagulation control. In this situation, the decision to start or continue a patient on VKA versus switching to one of the new anticoagulants is an important and difficult one. Moreover, actual knowledge of novel oral anticoagulants is limited, particularly as these apply to elderly patients with multiple comorbid conditions, malnourished and suffering from chronic kidney failure. After 55 years of use, we are still learning about VKA, and novel oral anticoagulants cannot be adapted for administration to every patient. Expertise concerning VKA and the factors that influence their balance is still needed.
