# Correction: Comorbidities against Quality Control of VKA Therapy in Non-Valvular Atrial Fibrillation: A French National Cross-Sectional Study

**DOI:** 10.1371/journal.pone.0128867

**Published:** 2015-05-18

**Authors:** The PLOS ONE Staff

The fourth author’s name is spelled incorrectly. The correct name is: Guillaume Chapelet.

Additionally, Dr. Gilles Berrut should be included in the author byline. He should be listed as the fifth author, and his affiliation is 1: Department of Geriatrics, EA 1156–12, Nantes University Hospital, Nantes, France.

The correct citation is: Rouaud A, Hanon O, Boureau A-S, Chapelet G, Berrut G, de Decker L (2015) Comorbidities against Quality Control of VKA Therapy in Non-Valvular Atrial Fibrillation: A French National Cross-Sectional Study. PLoS ONE 10(3): e0119043. doi:10.1371/journal.pone.0119043

The publisher apologizes for the error.
